# The relationship between air quality and respiratory pathogens among children in Suzhou City

**DOI:** 10.1186/s13052-019-0702-2

**Published:** 2019-09-23

**Authors:** Dandan Zhang, Yuqin Li, Qiu Chen, Yanqun Jiang, Chu Chu, Ying Ding, Yixue Yu, Yujie Fan, Jinjin Shi, Yali Luo, Weifang Zhou

**Affiliations:** 1 Department of Infectious Disease, Children’s Hospital of Soochow University, Suzhou, 215003 China; 2 School of Radiation Medicine and Protection, Soochow University, Suzhou, 215123 China

**Keywords:** Air quality, Respiratory tract infection, Pathogens, Children, Model selection

## Abstract

**Objective:**

We studied the short-term effects of air pollutant concentrations in Suzhou City on respiratory infections in children of different age groups.

**Methods:**

We employed clinical data from children hospitalized with respiratory infections at the Children’s Hospital of Soochow University during 2014–2016, and air quality for Suzhou City covering the same period.We investigated the relationships between the air pollutant concentrations and respiratory tract infections in children by causative pathogen using time series models with lagged effects.

**Results:**

The results of single-pollutant models showed that PM2.5, PM10, NO_2_, SO_2_ and CO had statistically significant associations with respiratory tract infections in children under 3 years, with the largest effect sizes at a lag of 3 weeks. Notably, the multi-pollutant model found PM2.5 was significantly associated with viral respiratory in children under 7 months, and bacterial respiratory infections in other age groups, while PM10 concentrations were associated with viral infections in preschool children.

**Conclusion:**

PM2.5, PM10 and NO_2_ are the main atmospheric pollutants in Suzhou. The associations between pollutant concentrations and viral and bacterial respiratory infections were stronger among children under 3 years than for older age group.s PM2.5 had the strongest influence on viral and *Mycoplasma pneumoniae* respiratory infections when multiple pollutants were tested together.

## Introduction

The impact of air pollution on respiratory health in China has recently received significant attention. Air pollutants have been associated with multiple adverse effects on human health; in particular respiratory and cardiovascular health [1]. Residents of different regions have variable sensitivity to potential health effects of air pollutants [2]. Acute respiratory tract infection (ARI) is a common infectious disease to which children are particularly susceptible. Viruses account for over 80% of respiratory infections in infants and young children, although *Mycoplasma pneumoniae* and chlamydia are also implicated [3]. Secondary bacterial infection can follow viral infection. Bacteria of the genus Mycoplasma are among the most common causative pathogens of respiratory infection in children, and incidence of such infections has increased in China in recent years [4]. The developmental, structural and physiological characteristics of a child’s respiratory system determine sensitivity to effects of atmospheric pollution [5]. Previous work has found a positive correlation between air pollutant concentrations and incidence of respiratory diseases in children [6–8]. Furthermore, inhalation of air pollutants can change the micro-ecology of the respiratory tract and increase the susceptibility of children to respiratory infections [9].

This study sought to establish the most appropriate statistical model to assess short-term associations between air pollutant concentrations and respiratory tract infections in children, and to test the susceptibility of different age groups to inform health risk assessments and development of public health policy responses.

## Materials and methods

### Materials

We employed hospitalization data collected from the Children’s Hospital of Soochow University during 2014–2016 meeting diagnostic criteria for ARI [4], and extracted variables including age, gender, disease diagnosis, date of visit, medical record number, residential address, and pathogens detected. Our analytic sample included 5487 children with ARI aged 1 month to 15 years registered as being resident in Suzhou. It comprised 3373 boys (61.5%) and 2114 girls (38.5%) (male-to-female ratio: 1.6:1).This study was conducted with the approval of the Institutional Human Ethical Committee of Children’s Hospital of Soochow University. An informed consent was obtained from all the subjects or guardians who participated in this study.

We also used air quality data from the same period provided by the Suzhou Environmental Protection Agency. Readings included fine particulate matter (PM2.5), inhalable particulate matter (PM10), nitrogen dioxide (NO_2_), sulfur dioxide (SO_2_), carbon monoxide (CO) and ozone (O_3_). Daily air pollutant concentrations were calculated as the mean per 24-h period, except for O_3_ which was calculated flow-weighted mean concentration per eight-hour period. Concentrations of air pollutants were expressed in μg/m^3^, except CO for which mg/m^3^ was used.

## Methods

Descriptive statistics were carried out with SPSS 21.0 statistical software, including mean, standard deviation, the 25th, 50th (median) and 75th percentiles, minimum and maximum. The correlation between air pollutants was analyzed by Spearman correlation analysis.

First, the basic model was constructed, without an air pollutant and with other covariates, including time, day of the week and public holidays. While meteorological factors were not controlled. Bayesian Information Criterion (BIC) was used to measure how well the model fifitted the data. The smallest BIC value indicates the preferred model. Air pollutants were included step by step into the model. If the BIC value was smaller when a new variable was introduced, the variable remained in the model. This was the Model Selection mode.

Next, Single-pollutant models were established, each model contained one type of air pollutant. The associations between air pollution and health effects can be described as exhibiting a hysteresis effect, in which risk of a given outcome is dependent on both current and past pollutant concentrations, and where exposure to air pollutants may demonstrate a lagged effect after one or more days [10]. Lag0 to lag4 were used to analyz the associations between single pollutant and incidence of different types of respiratory tract infections (viral, Mycoplasma or other bacteria) among children of different age groups (1 month≤ age1 ≤ 6 months, 6 months< age2 ≤ 1 year, 1 year<age 3 ≤ 3 years, 3 years <age 4 ≤ 6 years, age 5 > 6 years). Here, lag0 corresponded to the current-day air pollutant concentration and lag1 referred to the average concentration of air pollutants in the week before admission and so on.

There is also the potential for interaction effects between various pollutants, however, and models fitting exposure to a single pollutant may underestimate the impact of atmospheric pollution on human health [11].Studies have shown differential susceptibility of children to different respiratory pathogens by age group [12, 13]. Considering the maximum effect of each air pollutant in the single-pollutant models, the air pollutant concentrations corresponding to the lag day were used to develop the multi-pollutant models.Based on the principle of BIC minimum value, the optimal model was selected to determine the main pollutants and sensitive period of different pathogens affecting children’s respiratory tract infection.

SPSS version 21.0 and SAS version 9.4 were used for data handling. All time series analyses were performed using R version 3.3.1. Results were expressed in terms of odd ratios (OR) for each additional 10 μg/m^3^ in atmospheric concentration with 95% confidence interval (95% CI), and *p*-values of < 0.05 were considered to indicate statistically significant associations.

## Results

### Characteristics of the air quality indicators

Mean annual concentrations of PM2.5 in Suzhou were 66.38 μg/m^3^ in 2014, 57.51 μg/m^3^ in 2015 and 45.41 μg/m^3^ in 2016. Annual mean PM10 concentrations were 89.94 μg/m^3^, 80.51 μg/m^3^ and 71.27 μg/m^3^ respectively. For NO_2_ these were 51.72 μg/m^3^, 50.85 μg/m^3^, and 48.71 μg/m^3^. Concentration of each of these pollutants exceeded the annual average secondary concentration limits established by the GB3095–2012 Ambient Air Quality Standard (PM2.5: 35 μg/m3, PM10: 70 μg/m^3^, NO_2_: 40 μg/m^3^). Mean concentrations of NO_2_, PM2.5 and PM10 were highest in winter (December, January and February) and decreased during the summer (June, July and August). The mean primary concentration limits for SO_2_, CO and O_3_ indicated by the GB3095–2012 standard are 20 μg/m^3^, 4 mg/m^3^ and 100 μg/m^3^. Atmospheric concentrations of SO_2_ in Suzhou exceeded the limit in 2014 and 2015 but not in in 2016. Annual mean concentrations of CO and O_3_ remained within the limit for the duration of the period studied. While concentrations of O_3_ are elevated in summer compared with winter, there was no significant monthly fluctuation in CO concentrations. These readings indicate that poor air quality in Suzhou City during 2014–2016 posed a risk to human health, and that concentrations of particulates were of notable concern (Table 1).

**Table 1:** Descriptive analysis air pollutants inSuzhou City, 2014–2016

	Pollutants	Mean	Sd	Min	P_25_	P_50_	P_75_	Max
2014	PM2.5 (μg/m^3^)	66.38	22.22	12	53	64	74	230
	PM10 (μg/m^3^)	89.94	26.25	14	72	87	102	283
	NO_2_ (μg/m^3^)	51.72	13.74	14	41	48	59	125
	SO_2_ (μg/m^3^)	23.06	7.68	5	16	19	24	60
	CO(mg/m^3^)	0.93	0.18	0.48	0.82	0.9	1.0	2.32
	O_3_ (μg/m^3^)	55.98	22.03	5	39	39	69	157
2015	PM2.5 (μg/m^3^)	57.51	23.40	7	41	53	69	230
	PM10 (μg/m^3^)	80.51	26.77	16	62	74	95	283
	NO_2_ (μg/m^3^)	50.58	14.37	13	40	50	62	125
	SO_2_ (μg/m^3^)	21.23	7.65	5	15	18	23	62
	CO(mg/m^3^)	0.93	0.21	0.43	0.77	0.88	1.05	2.32
	O_3_ (μg/m^3^)	57.44	23.63	5	36	62	75	157
2016	PM2.5 (μg/m^3^)	45.41	22.04	6	28	40	58	157
	PM10 (μg/m^3^)	71.27	24.96	10	53	67	86	281
	NO_2_ (μg/m^3^)	48.71	13.91	17	40	47	56	123
	SO_2_ (μg/m^3^)	16.84	5.30	5	12	14	18	39
	CO(mg/m^3^)	0.94	0.19	0.51	0.82	0.92	1.04	1.81
	O_3_ (μg/m^3^)	60.07	22.86	4	40	57	78	151

There were statistically significant differences in mean concentrations of PM2.5, PM10, NO_2_ and SO_2_ by year (*p* < 0.05), with higher concentrations of PM2.5, PM10, NO_2_ and SO_2_ in 2014 than in 2015 and 2016. (Table 2).

**Table 2:** Annual average concentration of air pollutants in Suzhou City in 2014–2016

Index	2014	2015	2016	F	*P*
PM2.5	66.38 ± 22.22	57.51 ± 23.40^ab^	45.41 ± 22.04^a^	97.54	< 0.001
PM10	89.94 ± 26.25	80.51 ± 26.77^ab^	71.27 ± 24.96^a^	51.4	< 0.001
NO_2_	51.72 ± 13.74	50.58 ± 14.37^b^	48.71 ± 13.91^a^	7.40	0.025
SO_2_	23.06 ± 7.68	21.23 ± 7.65^ab^	16.84 ± 5.30^a^	72.69	< 0.001
CO	0.93 ± 0.18	0.93 ± 0.21	0.94 ± 0.19	3.03	0.220
O_3_	55.98 ± 22.03	57.44 ± 23.63	60.07 ± 22.86	4.07	0.131
Number of patients	1959	1812^a^	1716^a^	16.56	< 0.001

Note: Compared with 2014, ^a^*p* < 0.05; compared with 2016, ^b^*p* < 0.05

We also found positive and statistically significant correlations between concentrations of PM2.5, PM10, NO_2_, SO_2_ and CO. There was a significant negative correlation between O_3_, PM2.5, NO_2_, SO_2_ and CO. (Table 3).

**Table 3:** Correlation between air pollutants

	PM2.5	PM10	NO_2_	SO_2_	CO	O_3_
PM2.5	1					
PM10	0.894**	1				
NO_2_	0.616**	0.600**	1			
SO_2_	0.690**	0.740**	0.643**	1		
CO	0.723**	0.643**	0.616**	0.544**	1	
O_3_	−0.137**	0.001	−0.470**	−0.183**	−0.343**	1

Note: *p < 0.05; **p < 0.01

### Characteristics of respiratory infections

During the period 2014–2016, nasopharyngeal peripheral blood samples were collected from 5487 children with ARI. Viral pathogens were detected in 1827 (33.3%) of these cases. The proportion of cases with a viral infection was higher in 2014 than in 2015 and 2016. These annual differences were statistically significant (χ^2^ = 43.80, *p* < 0.001). *M. pneumoniae* was detected in 1581 cases (28.8%) overall, including 542 (27.6%), 537 (29.6%), and 502 (29.2%) in 2014, 2015 and 2016 while 1657 other bacterial infections (30.2%) were detected with 629 (32.1%), 539 (29.7%), and 502 (29.2%) in each year (Table 4).

**Table 4:** Detection of pathogens of respiratory infections in 2014–2016

Pathogens	2014 *n* = 1959	2015 *n* = 1812	2016 *n* = 1716	Total *n* = 5487	*xef* ^2^	*P*-value
virus	780(39.8%)	535(29.5%)^a^	512(29.8%)^a^	182(33.3%)	43.80	< 0.001
bacteria	629(32.1%)	539(29.7%)	502(29.2%)	165(30.2%)	2.45	0.121
Mycoplasma	542(27.6%)	537(29.6%)	502(29.2%)	158(28.8%)	3.74	0.058

Note: Compared with 2014, ^a^*p* < 0.05

In the same period, our sample children aged 1 month to 15 years hospitalized for ARI included 2079 cases (37.9%) aged less than or equal to 6 months old, 753 (13.7%) aged 6 months to 1 year, 1262 (23%) aged 1 year to 3 years, 937 (17.1%) aged 3 years to 6 years, and 456 (8.3%) aged greater than 6 years (Table 5).

**Table 5:** Age stratification of children with respiratory infection

Year	Age 1	Age 2	Age 3	Age 4	Age 5
2014	763	281	474	289	152
2015	693	284	385	309	141
2016	623	188	403	339	163
Total	2,079(37.9%)	753(13.7%)	1,262(23%)	937(17.1%)	456(8.3%)

### Lagged associations between air quality and respiratory tract infections attributable to different pathogens in children by age group in single-pollutant model

#### Relationship between air quality and viral respiratory tract infections

We found significant associations between concentrations of PM2.5, PM10, NO_2_, SO_2_ and CO at lag 0–lag 4 and incidence of viral respiratory tract infections in children under 3 years of age in Suzhou City during 2014–2016 (*p* < 0.05), with the largest effect size found for lag 3. The association between NO_2_ and incidence of viral respiratory infections in preschool children was statistically significant (*p* < 0.05), with the largest effect size found at lag 2. No such associations were found for PM2.5, PM10, SO_2_ and CO, however (*p* > 0.05). There was also a significant association between SO_2_ concentrations and viral respiratory infections in school-age children (< 0.05), with the largest effect size at lag 4. There was no significant association found for PM2.5, PM10, NO_2_ or CO (*p* > 0.05). We also found a negative association between O_3_ concentrations at lag 0–lag 4 and viral respiratory infections in children under 3 years (*p* < 0.05) (Table 6).

**Table 6:** Hysteresis effect of air quality on respiratory tract infection virus in children in age group

Age	Pollutants	Lag 0		Lag 1		Lag 2		Lag 3		Lag 4	
		OR	95%CI	OR	95%CI	OR	95%CI	OR	95%CI	OR	95%CI
Age 1	PM2.5	1.005	1.002–1.008	1.009	1.005–1.014	1.012	1.008–1.018	1.013	1.008–1.018	1.011	1.006–1.016
	PM10	1.003	1.001–1.006	1.006	1.002–1.010	1.009	1.005–1.014	1.010	1.006–1.015	1.009	1.005–1.014
	NO_2_	1.020	1.015–1.026	1.033	1.025–1.040	1.039	1.031–1.047	1.045	1.036–1.053	1.044	1.035–1.053
	SO_2_	1.039	1.028–1.049	1.059	1.045–1.072	1.068	1.054–1.083	1.070	1.055–1.085	1.066	1.051–1.081
	CO	2.598	1.857–3.634	11.307	6.535–19.563	28.952	15.453–44.244	40.146	20.978–59.315	35.576	16.467–54.686
	O_3_	0.979	0.974–0.983	0.968	0.963–0.973	0.967	0.962–0.973	0.967	0.962–0.972	0.968	0.963–0.973
Age 2	PM2.5	1.004	0.999–1.009	1.010	1.004–1.018	1.010	1.003–1.018	1.011	1.003–1.019	1.011	1.003–1.019
	PM10	1.002	0.998–1.006	1.007	1.001–1.012	1.007	1.000–1.014	1.009	1.002–1.017	1.009	1.000–1.015
	NO_2_	1.012	1.004–1.020	1.030	1.020–1.044	1.031	1.019–1.045	1.032	1.017–1.043	1.029	1.015–1.042
	SO_2_	1.022	1.005–1.039	1.049	1.027–1.070	1.051	1.029–1.075	1.052	1.027–1.077	1.052	1.027–1.076
	CO	2.060	1.008–3.111	5.385	1.698–9.020	6.076	2.411–9.712	12.140	2.936–21.273	11.715	3.254–20.177
	O_3_	0.985	0.979–0.991	0.978	0.971–0.986	0.976	0.968–0.984	0.978	0.971–0.986	0.979	0.971–0.987
Age3	PM2.5	1.006	1.002–1.011	1.012	1.005–1.019	1.012	1.005–1.021	1.013	1.005–1.021	1.012	1.004–1.020
	PM10	1.005	1.001–1.009	1.010	1.004–1.016	1.011	1.004–1.019	1.012	1.004–1.020	1.011	1.004–1.020
	NO_2_	1.016	1.008–1.024	1.025	1.013–1.037	1.027	1.015–1.041	1.031	1.015–1.047	1.029	1.015–1.045
	SO_2_	1.042	1.026–1.058	1.056	1.034–1.078	1.056	1.034–1.080	1.059	1.035–1.083	1.058	1.035–1.082
	CO	2.717	1.356–4.078	9.770	2.949–16.590	13.575	3.479–23.671	19.182	4.414–33.950	17.478	4.038–30.917
	O_3_	0.989	0.983–0.995	0.983	0.976–0.991	0.981	0.973–0.989	0.980	0.972–0.988	0.979	0.971–0.987
Age 4	PM2.5	1.004	0.996–1.011	1.000	0.991–1.011	1.001	0.991–1.012	1.001	0.988–1.011	0.999	0.988–1.011
	PM10	1.002	0.996–1.008	0.999	0.990–1.009	0.999	0.989–1.009	0.999	0.986–1.008	0.995	0.984–1.007
	NO_2_	1.016	1.004–1.028	1.024	1.008–1.041	1.025	1.007–1.043	1.024	0.999–1.038	1.015	0.995–1.036
	SO_2_	1.018	0.994–1.043	1.023	0.990–1.056	1.018	0.984–1.053	1.018	0.977–1.052	1.007	0.968–1.047
	CO	1.823	0.810–4.101	1.257	0.361–4.381	1.478	0.380–5.752	1.478	0.421–8.195	2.032	0.454–9.092
	O_3_	0.989	0.980–0.998	0.992	0.982–1.003	0.993	0.982–1.004	0.993	0.980–1.003	0.989	0.978–1.001
Age 5	PM2.5	1.008	0.997–1.019	1.012	0.994–1.032	1.016	0.996–1.039	1.021	0.999–1.043	1.021	0.999–1.042
	PM10	1.002	0.992–1.013	1.010	0.994–1.026	1.011	0.992–1.031	1.016	0.995–1.036	1.018	0.998–1.039
	NO_2_	1.000	0.975–1.025	1.028	0.999–1.058	1.031	0.998–1.066	1.033	0.998–1.070	1.035	0.999–1.073
	SO_2_	1.039	0.997–1.083	1.052	0.999–1.108	1.062	1.005–1.123	1.072	1.014–1.133	1.085	1.024–1.146
	CO	2.863	0.751–10.915	9.244	0.951–89.866	10.963	0.812–147.987	10.893	0.701–169.223	12.177	0.764–194.023
	O_3_	0.986	0.969–1.003	0.981	0.961–1.001	0.981	0.961–1.002	0.976	0.955–0.998	0.985	0.954–0.997

#### Relationship between air quality and bacterial respiratory tract infections

We found significant associations between PM2.5, PM10, NO_2_ and SO_2_ concentrations at lag 0–lag 4 and bacterial respiratory tract infections among children less than or equal to 6 months of age during 2014–2016 (*p* < 0.05). The largest effect sizes were found for lag 4. Associations between concentrations of PM2.5, PM10 and SO_2_ at lag 0–lag 4 and respiratory tract infections in children aged 6 months to 3 years were also statistically significant (*p* < 0.05), with the largest effect size at lag 4. We found PM10 and SO_2_ concentrations were significantly associated with infections in preschool children (*p* < 0.05). The largest effect sizes for PM10 and SO_2_ were at lag 4 and lag 1. No significant associations were found in school-age children, however (*p* > 0.05). While there was a significant association between O_3_ concentration at any lag and incidence of bacterial respiratory infection in children less than or equal to 6 months (*p* < 0.05), no significant associations were found for children in other age groups (*p* > 0.05). (Table 7).

**Table 7:** Hysteresis effect of air quality on bacteria in respiratory tract infection in children in age group

Age	Pollutants	Lag 0		Lag 1		Lag 2		Lag 3		Lag 4	
		OR	95%CI	OR	95%CI	OR	95%CI	OR	95%CI	OR	95%CI
Age1	PM2.5	1.004	1.002–1.007	1.010	1.006–1.014	1.012	1.007–1.016	1.013	1.009–1.018	1.014	1.010–1.019
	PM10	1.004	1.002–1.006	1.010	1.006–1.013	1.011	1.007–1.016	1.015	1.010–1.019	1.016	1.011–1.020
	NO_2_	1.004	1.004–1.014	1.008	1.002–1.015	1.007	1.000–1.014	1.008	1.002–1.017	1.009	1.001–1.017
	SO_2_	1.028	1.018–1.038	1.050	1.037–1.063	1.049	1.035–1.063	1.050	1.035–1.065	1.051	1.036–1.065
	CO	1.110	0.815–1.510	1.130	0.697–1.833	0.988	0.575–1.698	1.278	0.633–2.009	0.937	0.514–1.708
	O_3_	0.995	0.992–0.999	0.994	0.990–0.998	0.995	0.991–1.000	0.995	0.991–1.000	0.996	0.991–1.000
Age2	PM2.5	0.999	0.995–1.003	1.007	1.001–1.013	1.008	1.002–1.015	1.008	1.002–1.015	1.009	1.002–1.015
	PM10	0.999	0.996–1.002	1.006	1.001–1.011	1.009	1.003–1.015	1.009	1.003–1.016	1.010	1.004–1.016
	NO_2_	1.002	0.995–1.009	1.008	0.998–1.017	1.005	0.995–1.016	1.005	0.994–1.016	1.005	0.993–1.016
	SO_2_	1.007	0.992–1.021	1.029	1.010–1.049	1.028	1.008–1.050	1.028	1.006–1.050	1.030	1.007–1.052
	CO	0.854	0.530–1.375	1.547	0.760–3.149	1.469	0.662–3.257	1.263	0.540–2.955	1.071	0.448–2.559
	O_3_	0.995	0.990–0.999	0.996	0.990–1.001	0.997	0.991–1.003	0.997	0.991–1.004	0.997	0.991–1.004
Age3	PM2.5	1.001	0.998–1.004	1.005	1.000–1.010	1.006	1.001–1.012	1.007	1.002–1.013	1.008	1.002–1.014
	PM10	1.001	0.998–1.004	1.004	0.999–1.008	1.006	1.001–1.011	1.008	1.003–1.014	1.009	1.003–1.015
	NO_2_	1.003	0.998–1.009	1.003	0.995–1.011	1.000	0.992–1.010	1.001	0.992–1.011	1.000	0.991–1.010
	SO_2_	1.009	0.997–1.022	1.017	1.001–1.033	1.020	1.003–1.038	1.022	1.004–1.041	1.024	1.006–1.042
	CO	1.025	0.685–1.532	0.789	0.427–1.456	0.564	0.281–1.130	0.597	0.285–1.250	0.586	0.276–1.241
	O_3_	0.995	0.992–0.999	1.000	0.995–1.005	1.003	0.998–1.008	1.004	0.998–1.009	1.004	0.998–1.009
Age4	PM2.5	1.001	0.999–1.007	1.001	0.998–1.010	1.006	1.000–1.012	1.006	1.000–1.013	1.006	1.000–1.013
	PM10	1.003	0.998–1.005	1.003	1.000–1.010	1.006	1.001–1.012	1.006	1.002–1.014	1.007	1.001–1.014
	NO_2_	1.007	0.995–1.010	1.007	0.998–1.017	1.006	0.995–1.017	1.006	0.991–1.013	0.999	0.988–1.011
	SO_2_	0.978	0.992–1.022	1.033	1.012–1.053	1.032	1.011–1.053	1.028	1.006–1.050	1.021	0.998–1.044
	CO	0.995	0.607–1.577	0.995	0.323–1.329	0.717	0.328–1.567	0.717	0.228–1.285	0.538	0.222–1.301
	O_3_	1.000	0.990–1.000	1.000	0.990–1.002	0.998	0.991–1.004	0.998	0.993–1.005	0.999	0.992–1.005
Age5	PM2.5	1.000	0.994–1.006	1.004	0.996–1.013	1.008	0.999–1.018	1.009	0.999–1.019	1.009	0.999–1.019
	PM10	0.999	0.994–1.004	1.004	0.996–1.012	1.009	1.000–1.018	1.010	1.000–1.019	1.009	1.000–1.019
	NO_2_	0.995	0.984–1.006	0.991	0.977–1.005	0.992	0.976–1.008	0.990	0.974–1.006	0.987	0.971–1.005
	SO_2_	1.001	0.979–1.023	1.010	0.983–1.038	1.017	0.987–1.047	1.016	0.986–1.047	1.015	0.985–1.047
	CO	0.633	0.307–1.306	0.422	0.132–1.350	0.483	0.128–1.829	0.447	0.112–1.789	0.345	0.080–1.480
	O_3_	0.994	0.987–1.001	1.002	0.993–1.010	1.005	0.996–1.014	1.006	0.996–1.016	1.006	0.996–1.017

#### Relationship between air quality and respiratory tract infections caused by mycoplasma pneumoniae

PM2.5, NO_2_, and SO_2_ concentrations were negatively associated with respiratory infections attributable to *M. pneumoniae* in children under 3 years. PM2.5 was also negatively associated with *M. pneumoniae* infections among children of pre-school age, while negative associations were found for both PM2.5 and PM10 in school-age children. There was a positive and statistically significant association between O_3_ concentrations at lag 0–lag 4 and *M. pneumoniae* respiratory tract infections in children under 6 years old (*p* < 0.05), with the largest effect size found for lag 4 (Table 8).

**Table 8:** Hysteresis effect of air quality on the respiratory tract infection of *Mycoplasma pneumoniae* in children in the subgroup of children

Age	Pollutants	Lag0		Lag1		Lag2		Lag3		Lag4	
		OR	95%CI	OR	95%CI	OR	95%CI	OR	95%CI	OR	95%CI
Age1	PM2.5	0.999	0.997–1.002	0.995	0.991–0.998	0.993	0.989–0.997	0.992	0.988–0.997	0.991	0.986–0.995
	PM10	1.001	0.999–1.003	1.000	0.996–1.003	0.999	0.995–1.003	0.998	0.994–1.002	0.996	0.992–1.001
	NO_2_	1.000	0.995–1.005	0.991	0.985–0.998	0.989	0.982–0.996	0.987	0.980–0.994	0.983	0.976–0.991
	SO_2_	1.004	0.995–1.013	0.992	0.981–1.003	0.985	0.973–0.998	0.979	0.967–0.992	0.974	0.961–0.987
	CO	1.017	0.750–1.380	0.740	0.458–1.196	0.644	0.376–1.104	0.533	0.300–0.946	0.622	0.232–0.770
	O_3_	1.003	1.000–1.006	1.007	1.003–1.011	1.008	1.004–1.012	1.009	1.005–1.014	1.010	1.005–1.014
Age2	PM2.5	0.995	0.991–1.000	0.992	0.986–0.998	0.991	0.984–0.997	0.989	0.982–0.996	0.988	0.981–0.995
	PM10	0.998	0.995–1.002	0.996	0.991–1.001	0.996	0.990–1.002	0.995	0.988–1.001	0.993	0.987–1.000
	NO_2_	0.991	0.984–0.998	0.987	0.977–0.997	0.988	0.977–0.998	0.986	0.975–0.997	0.983	0.971–0.994
	SO_2_	0.991	0.977–1.007	0.984	0.966–1.002	0.982	0.963–1.002	0.974	0.954–0.995	0.971	0.951–0.992
	CO	0.797	0.485–1.311	0.683	0.327–1.426	0.627	0.275–1.429	0.472	0.196–1.139	0.387	0.158–0.952
	O_3_	1.004	0.999–1.009	1.007	1.001–1.013	1.008	1.002–1.015	1.009	1.003–1.016	1.011	1.004–1.019
Age3	PM2.5	0.999	0.995–1.003	0.991	0.986–0.997	0.989	0.983–0.995	0.986	0.980–0.993	0.986	0.980–0.993
	PM10	1.000	0.997–1.003	0.995	0.990–1.000	0.993	0.987–0.999	0.997	0.985–0.997	0.996	0.985–0.997
	NO_2_	0.998	0.991–1.004	0.989	0.980–0.998	0.983	0.973–0.993	0.980	0.970–0.990	0.978	0.967–0.988
	SO_2_	0.998	0.985–1.012	0.986	0.968–1.003	0.978	0.960–0.996	0.970	0.952–0.989	0.967	0.948–0.985
	CO	1.092	0.687–1.736	0.924	0.460–1.854	0.810	0.370–1.773	0.560	0.245–1.284	0.452	0.197–1.041
	O_3_	1.004	1.000–1.009	1.007	1.002–1.013	1.009	1.004–1.015	1.011	1.004–1.017	1.012	1.006–1.018
Age4	PM2.5	0.996	0.991–1.001	0.991	0.985–0.998	0.989	0.982–0.996	0.989	0.978–0.994	0.985	0.978–0.993
	PM10	1.000	0.996–1.004	0.997	0.991–1.003	0.994	0.988–1.001	0.994	0.984–0.999	0.991	0.984–0.998
	NO_2_	1.000	0.991–1.010	0.996	0.984–1.008	0.992	0.979–1.004	0.992	0.975–1.002	0.985	0.972–1.000
	SO_2_	0.999	0.981–1.018	0.990	0.967–1.013	0.984	0.960–1.008	0.984	0.951–1.001	0.970	0.945–0.996
	CO	1.338	0.728–2.460	1.110	0.462–2.671	0.717	0.278–1.850	0.717	0.230–1.851	0.601	0.209–1.732
	O_3_	1.003	0.997–1.009	1.007	1.001–1.013	1.010	1.003–1.018	1.010	1.003–1.019	1.012	1.003–1.020
Age5	PM2.5	0.995	0.988–1.001	0.987	0.976–0.997	0.985	0.973–0.997	0.984	0.972–0.996	0.985	0.973–0.997
	PM10	0.997	0.991–1.003	0.988	0.979–0.998	0.988	0.978–0.999	0.985	0.974–0.997	0.985	0.974–0.997
	NO_2_	1.001	0.988–1.015	0.984	0.967–1.000	0.982	0.964–1.001	0.982	0.963–1.002	0.983	0.963–1.004
	SO_2_	1.003	0.975–1.033	0.985	0.953–1.018	0.982	0.947–1.018	0.977	0.942–1.013	0.973	0.938–1.009
	CO	0.750	0.322–1.747	0.575	0.144–2.291	0.538	0.110–2.646	0.589	0.112–3.105	0.646	0.115–3.620
	O_3_	1.000	0.991–1.009	0.999	0.988–1.009	0.998	0.986–1.009	0.996	0.984–1.009	0.997	0.984–1.010

#### Analysis of multi-pollutant effects on the risk of respiratory infections in children by age subgroups and model selection

The multi-pollutant model showed that after mutual adjustment for effects of other air pollutants, there were significant associations between both PM2.5 and O_3_ concentrations and viral respiratory infections in children less than or equal to 6 months (*p* < 0.05) with ORs of 1.019 (95% CI: 1.012–1.026) and 1.025 (95% CI: 1.018–1.033) for each additional 10 μg/m^3^. The associations between O_3_ and viral respiratory infections in children aged 6 months to 3 years was also statistically significant (OR: 1.025, 95% CI: 1.016–1.033, *p* < 0.05), and PM10 and viral respiratory infections virus in preschool children (OR: 1.025, 95% CI: 1.008–1.042, *p* < 0.05) were both statistically significant but had similar effect sizes to the single pollutant model. When included in the same multi-pollutant model, negative associations were found between SO_2_ and NO_2_ concentrations and viral infections across all age groups (Table 9).

**Table 9:** Effect of multiple pollutants on the risk of viral respiratory infection in the Model Selection

Age	Pollutants	OR	95%CI	*P*
Age1	PM2.5	1.019	1.012–1.026	< 0.001
	SO_2_	0.950	0.929–0.972	< 0.001
	O_3_	1.025	1.018–1.033	< 0.001
Age2	NO_2_	0.970	0.953–0.988	0.001
	O_3_	1.025	1.016–1.033	< 0.001
Age3	SO_2_	0.966	0.950–0.981	< 0.001
	O_3_	1.007	1.001–1.014	0.021
Age4	PM10	1.025	1.008–1.042	0.003
	NO_2_	0.940	0.912–0.970	< 0.001
Age5	SO_2_	0.923	0.873–0.977	0.006

We found a significant relationship between NO_2_ and bacterial respiratory tract infections in children less than or equal to 6 months (OR: 1.050, 95% CI: 1.029–1.072, *p* < 0.05) with a larger effect size than in the single-contaminant model. The results of the same model show inverse associations for PM2.5, PM10, and SO_2_. Meanwhile, the association between O_3_ and infections in children aged 6 months to 6 years was also statistically significant (OR: 1.005, 95% CI: 1.001–1.010, *p* < 0.05). Similar associations and effect sizes were found for other age groups (Table 10).

**Table 10:** Effect of multiple pollutants on the risk of bacterial respiratory infections in the Model Selection

Age	Pollutants	OR	95%CI	*P*
Age1	PM2.5	0.899	0.866–0.933	< 0.001
	PM10	0.969	0.953–0.986	< 0.001
	NO_2_	1.050	1.029–1.072	< 0.001
	SO_2_	0.865	0.832–0.900	< 0.001
Age2	O_3_	1.005	1.001–1.010	0.020
	PM10	0.980	0.971–0.989	< 0.001
Age3	O_3_	1.005	1.001–1.010	0.020
	PM10	0.980	0.971–0.989	< 0.001
Age4	PM10	0.980	0.971–0.988	< 0.001
	O_3_	1.016	1.005–1.027	0.004

Except for O_3_, which was negatively associated with *M. pneumoniae* respiratory infections in children less than or equal to 6 months of age, there were no statistically significant associations found for the remaining pollutants. We found a significant association between PM2.5 and *M. pneumoniae* infections in children in all four age groups over 6 months (*p* < 0.05), with ORs of 1.021 (95% CI: 1.007–1.034), 1.048 (95% CI: 1.029–1.067), 1.067 (95% CI: 1.042–1.094) and 1.025 (95% CI: 1.010–1.041). The mutually-adjusted association between PM10 and *M. pneumoniae* infections was statistically significant for children in aged 6 months to 1 year and greater than 6 years (*p* < 0.05), with ORs of 1.029 (95% CI: 1.013–1.046) and 1.015 (95% CI: 1.002–1.019). The effect of NO_2_ on the incidence of respiratory infection with *M. pneumoniae* was statistically significant (*p* < 0.05), and the corresponding OR value was 1.048 (95% CI: 1.025–1.071). The mutually-adjusted association between SO_2_ and *M. pneumoniae* infection in school-age children (Age 5) was also statistically significant (OR: 1.016, 95% CI: 1.001–1.031, *p* < 0.05) (Table 11).

**Table 11:** Effect of multiple pollutants on the risk of respiratory infections of Mycoplasma pneumoniae in the Model Selection

Age	Pollutants	OR	95% CI	*P*
Age1	O_3_	0.991	0.987–0.995	< 0.001
Age2	PM2.5	1.021	1.007–1.034	0.003
	PM10	1.029	1.013–1.046	< 0.001
Age3	PM2.5	1.048	1.029–1.067	< 0.001
	PM10	0.964	0.947–0.982	< 0.001
	NO_2_	1.048	1.025–1.071	< 0.001
Age4	PM2.5	1.067	1.042–1.094	< 0.001
	PM10	0.961	0.939–0.983	< 0.001
Age5	PM2.5	1.025	1.010–1.041	0.001
	PM10	1.015	1.002–1.029	0.027
	SO_2_	1.016	1.001–1.031	0.036

## Discussion

This study investigated the relationship between different air pollutants and respiratory infections in children of different age groups over a three-year period, and used model selection to identify optimal models with mutual adjustment for multiple pollutants based on the BIC.

Air pollution, which not only has adverse ecological effects but also results in direct and indirect harm to human health, has become an increasingly prominent public health issue as China’s economy has developed. At present, most Chinese research on atmospheric pollution and its public health effects has concentrated on populations of megacities or heavily polluted areas. Contexts where pollution exposure is moderate, however, have received less attention. The air quality and environment of Suzhou City have gradually deteriorated in recent years due to accelerating development.

While viral infections are most common infants and young children, respiratory infections in preschool and school-age children are often bacterial [14]. The reported annual incident of bacterial respiratory infections in Chinese children is 10.90–56.82% [15], while incidence of *M. pneumoniae* infections has increased in recent years to reach 15–20% [16, 17]. This study retrospectively analyzed the pathogens found in nasopharyngeal samples and *M. pneumoniae* antibodies detected in peripheral blood in hospitalized children with ARIs. Among them, 1827 specimens, or 33.3% of our sample, tested positive for viral infections This is lower than in analytic samples in Tianjin, 74.8% [18] and Vietnam 69.0% [19], but higher than in a study in Zhejiang 22.0% [20]. Furthermore, 1657 (30.2%) cases tested positive for bacterial infections (excluding *M. pneumoniae*). This proportion is higher than in another study in Suzhou (26.3%) [21] but lower than reported in in central and southern Sichuan (34.6%) [22]. We found 28.8% of cases tested positive for *M. pneumoniae.* This proportion was lower than that reported in Shenyang [23] but higher than in another study based in Suzhou (19.0%) [24].

The atmosphere represents a medium through which microbes proliferate and transmit infection. One study found that air pollution can change the balance of respiratory flora in children, in particular a reduction in commensal *a-hemolytic streptococcus*, which can lead to an increase in other abnormal flora and increase the susceptibility to respiratory diseases [25]. This may represent a mechanism through which the air pollutants tested in our study may have influenced the incidence of respiratory infections in our study population.

### Effects of air pollutants on viral and bacterial respiratory tract infections in children of different ages

The results of the single-contaminant models showed that PM2.5, PM10, NO_2_, SO_2_, and CO had stronger associations with viral and bacterial respiratory infections children under 3 years of age than in older children. This may be due to the faster respiratory rate of infants and young children, immune system immaturity, and lower capacity to synthesize antibodies; all of which may lead to greater susceptible to air pollutants. More advanced development of immune system function in preschool and school-age children may reduce the probability of respiratory infections. A study of the short-term association between ambient air pollution and daily hospitalization for respiratory infections in children aged 0–17 years in Hanoi, Vietnam found that all ambient air pollutants tested were positively associated with hospitalization for pneumonia and nearly all were positively associated with bronchitis and hospitalization for asthma [26]. These associations were stronger for infants and young children than for those aged 3–5 years. Another study based in the United States tested the effects of short-term changes in air pollutant concentrations on the number of hospital visits attributed to respiratory infections in children aged 0–4 years. The results showed that O_3_ had the strongest association, and that the rate of hospital visits was higher in the preschool group than in infants [27]. Similar to the results of the single-contaminant models in this study, air pollutants are more strongly associated with viral and bacterial respiratory in children under 3 years of age than in older children. We also found that concentrations of air pollutants at lags of three and 4 weeks had the greatest effect sizes Toxicological studies have shown that the effects of air pollutants on human health have a physiological lag period [28]. A large population-based study found an association between the number of emergency room visits by children under 5 years of age for respiratory infections and short-term increases in O_3_ and NO_2_ concentrations with a lag of 3 days. Other literature suggests that the length of this lag period may be greater for children under 5 years of age than for older children [29]. These lag periods have been found to differ in length between studies, and this variability may be related to differences in the characteristics of their study populations, research designs, modeling strategies, pollutant measurement standards, and whether they investigated potential confounding factors. After all air pollutants were considered for inclusion in the multi-pollutant model as part of our model selection procedure, negative associations were found between SO_2_ and NO_2_ and respiratory tract infections while there was a positive association for O_3_. PM2.5, PM10 and SO_2_ concentrations were negatively associated with respiratory tract infections in children while a positive association was found for O_3_. The strength of the association between NO_2_ concentrations and infections increased with age. This may be due correlation in concentrations of different air pollutants, SO_2_ attaching to PM2.5, the fact that toxicity of pollutants is greater when they penetrate into the lower respiratory tract, and the negative correlation between the concentration of O_3_ and polycyclic aromatic hydrocarbons. As a result, when different pollutants interact, their potential effects on incidence of respiratory tract infections may be strengthened or weakened. This may have explained the inconsistencies between the results of our single pollutant and multi-pollutant models.

A study in Shenzhen [30] found that short-term exposure to ambient air pollution was significantly associated with hospitalization for ARIs. Children under 14 years of age were identified as most susceptible population to infections attributed to PM2.5, PM10 and NO_2_ pollution. An Iranian study has concluded that atmospheric particulate matter, NO_2_ and O_3_ are significantly associated with death by respiratory causes, and that these associations are subject to long lag times [31]. Although the findings of these studies may not be fully consistent to our own, this may be due to differences in pollutant monitoring, air pollutant composition and statistical methods employed.

### Effects of air pollutants on respiratory tract infections in children of different ages in our study

The results of single-pollutant models showed significant associations between O_3_ concentations and respiratory tract infections with *M. pneumoniae* in children under 6 years old, with exposure at a lag of 4 weeks having the greatest effect size. In contrast to the single-contaminant model, the results of the multi-pollutant model show that O_3_ was negatively associated with *M. pneumoniae* infection in children with aged 6 months and older; although this association was not significant for the remaining age groups. *M. pneumoniae*is a common cause of respiratory infection in children, and its incidence has increased in recent years. Although incidence of Mycoplasma infection tends to peak among school-age children, its mean age of first occurrence has risen in China. Animal experiments show that the pathological changes appear in tissues 1–2 weeks after initial infection with *M. pneumoniae*. When reinfection occurs, these changes usually appear within 3 days after infection. This suggests that the immune response has strengthened following the first infection. Following infection, B lymphocytes can be activated to produce specific antibodies including lgM, lgG and IgA. IgM, which is considered a marker of acute infection, appears more than 1 week after infection, and peaks at 3–4 weeks. While concentrations of IgA and IgM fall shortly afterwards, IgG production peaks later, and concentrations remain elevated for several months [32]. The associations between O_3_ and *M. pneumoniae* infections in children found by both the single contaminant and multi- contaminant models differ from those found in other countries. Bono et al. [33] have concluded that, based on the results of a single-contaminant model, O_3_ concentrations were associated with higher numbers of emergency room visits by children for respiratory problems. A study in Hefei City on the relationship between air pollution and upper respiratory tract infection in children aged 0–14 years during 2014–2015 found that O_3_ was significantly associated with incidence of infection when tested using a single pollutant model, while only NO_2_ was associated with infections when tested using a multi-pollutant model [29]. This difference may be due to the fact that O_3_ concentrations in this study did not exceed levels associated with harm to human health. O_3_ is considered a secondary pollutant, and, as such, is effect may be masked when present alongside other primary pollutants. Inconsistencies may also appear when comparing results based on tests of nasopharyngeal samples and serum antibody tests. In addition, most *M. pneumoniae* infections in infants and young children may be due to contact infected family members; particularly older family members who may have more frequent contacts with infected people outside the household. In addition, air pollutants are more strongly associated with *M. pneumoniae* infections in older people than young children. Further consideration of the susceptibility of the wider population, the individual characteristics of the children included in the analytic sample and the composition of air pollution at a regional level would be needed to gain a fuller picture of the determinants of respiratory infections in our study population.

Controlling air pollution remains a significant public health challenge in China. Our results highlight the need for interventions to reduce air pollution at the same time as strengthening the prevention, diagnosis and treatment of respiratory infections in children.

### Future research directions

The objective of this study was to identify the most appropriate statistical model for testing the relationship between air quality and respiratory tract infections in children of different ages. We did not adjust for individual factors (such as physical fitness, immune function, and health behaviors) or factors relating to the home environment (such as exposure to passive smoking). Future work on the associations between environmental pollutants and childhood respiratory infections should consider individual family factors to adjust for potential confounding.

## Conclusions

Our key finding from the single-pollutant models was that air pollutant concentrations had stronger associations with viral and bacterial respiratory infections in children under 3 years than in children older than 3 years. Concentrations of air pollutants at three- and four-week lags showed the strongest effects on respiratory tract infections in infants. When we tested multiple pollutants simultaneously, PM2.5 had the strongest influence on viral and *M. pneumoniae* respiratory tract infections.

Finally, the single-contaminant model showed that O_3_ was positively associated with respiratory infections in children under 6 years of age with the greatest effect size at a lag of 4 weeks. The multi-pollutant model showed a positive association between O_3_ and respiratory infections after mutual adjustment for other pollutants.

## Data Availability

All data is available.
